# Incidental thyroid carcinoma in an endemic goiter area in Italy: histopathological features and predictors of a common finding

**DOI:** 10.1007/s12020-023-03659-2

**Published:** 2024-01-13

**Authors:** Eusebio Chiefari, Nadia Innaro, Rita Gervasi, Maria Mirabelli, Stefania Giuliano, Alessandra Donnici, Stefania Obiso, Francesco S. Brunetti, Daniela Patrizia Foti, Antonio Brunetti

**Affiliations:** 1 Department of Health Sciences, University of “Magna Græcia” Catanzaro, 88100 Catanzaro, Italy; 2 Operative Unit of Endocrine Surgery, University Hospital “Renato Dulbecco”, 88100 Catanzaro, Italy; 3 Operative Unit of Endocrinology, University Hospital “Renato Dulbecco”, 88100 Catanzaro, Italy; 4 Department of Experimental and Clinical Medicine, University of “Magna Græcia” Catanzaro, 88100 Catanzaro, Italy

**Keywords:** Incidental thyroid carcinoma, Microcarcinoma, Papillary thyroid carcinoma, Goiter, Total thyroidectomy

## Abstract

**Purpose:**

The occurrence and histopathological features of incidental thyroid carcinoma (ITC) vary considerably among populations from different geographical regions. The aim of this study is to assess the prevalence and histopathological characteristics of ITC in patients who underwent thyroid surgery for apparently benign thyroid diseases in an endemic goiter area in Italy.

**Methods:**

A total of 649 consecutive patients (531 females and 118 males; mean age, 52.9 ± 11.0 years), who underwent thyroid surgery at the Endocrine Surgery Unit of the tertiary care “Renato Dulbecco” University Hospital (Catanzaro, Italy) in the period between years 2017 and 2022, were included in this retrospective study. A comprehensive histopathological examination was performed on surgically excised thyroid tissue. Logistic regression analysis was employed to identify potential predictors of ITC.

**Results:**

The histopathological examination revealed the presence of ITC in 81 patients, accounting for 12.5% of the total study population. The female to male ratio was found to be 6.4 to 1. Among the patients with ITC, 72 had papillary carcinoma (PTC), with 53 of these tumors being microcarcinomas (microPTC). Additionally, 5 patients had follicular thyroid carcinoma, 2 patients had low-risk follicular cell-derived thyroid neoplasms, 1 patient had an oncocytic carcinoma, and 1 patient had a medullary thyroid carcinoma. Logistic regression analysis demonstrated a significant association between female sex and incidental microPTC.

**Conclusions:**

These findings provide further evidence of the common occurrence of ITC, typically in the form of microPTC, among individuals who undergo thyroid surgery for apparently benign thyroid diseases.

## Introduction

Thyroid nodular disease is highly prevalent, particularly in regions with a deficiency of iodine, where it can affect more than half of adult women [1]. Nodules can manifest as single or multiple lesions, may become autonomously functioning and/or may lead to local compression symptoms such as dyspnea and/or dysphagia. Additionally, a small proportion (approximately 5%) of thyroid nodules are malignant [1].

The prevalence of thyroid cancer has significantly increased in the past few decades globally, although the rates of increase vary among different populations [2]. It has been observed that this rapid increase specifically applies to papillary thyroid cancer (PTC), which is the most common subtype of thyroid carcinoma and is often discovered incidentally in an indolent form [3]. Various factors, including iodination programs in areas with low iodine intake, ultrasonographic screening in regions with a high prevalence of goiter, the growing use of fine-needle aspiration biopsy (FNAB), and the thorough examination of the removed thyroid tissue, as well as environmental factors like obesity, have been linked to the rising rates of both micro (maximum diameter ≤10 mm) and macro (maximum diameter >10 mm) PTC [4–7]. MicroPTCs account for approximately half of all thyroid malignancies. In the majority of cases, microPTCs demonstrate a slow-growing nature and do not necessitate aggressive therapeutic intervention or extensive monitoring [8–11]. Nonetheless, there are instances where they may serve as precursors of more aggressive malignancies, and failure to promptly address these tumors can pose a significant risk to patient’s life, similarly to larger forms of thyroid cancer [12]. A wide portion of microPTCs, as well as larger forms of thyroid cancer, are incidentally discovered during thyroid surgery as they resemble benign conditions. The occurrence of these incidental thyroid carcinomas (ITC) varies from 3% to 16%, with some studies reporting rates exceeding 25% [13–16]. Despite the generally benign course of most microPTCs, certain studies highlight the biological aggressiveness of these small thyroid tumors, leading to the development of metastases, particularly in patients with Graves’ disease [17]. Furthermore, numerous studies have demonstrated that the likelihood of malignancy in a single thyroid nodule (SNG) is greater compared to a thyroid nodule in the context of a multinodular goiter (MNG), particularly in areas characterized by insufficient iodine levels [18–22], such as the Calabria region of southern Italy [23]. Despite the advancements in iodine nutrition witnessed over the past two decades, which can be attributed to the adoption of a nationwide program of iodine prophylaxis with iodized salt, as well as the establishment of a dedicated epidemiological observatory, it is notable that the rural and inland areas of Calabria still record a considerable prevalence of goiter in the adult population, estimated at 13.8% as of 2015 [23].

The aim of this study was to examine the prevalence, predictors and histopathological features of thyroid carcinomas that were unintentionally discovered in a series of patients from an endemic goiter area in Italy, who underwent thyroid surgery for an apparently benign thyroid disease.

## Materials and methods

### Study population

**Fig. 1:**
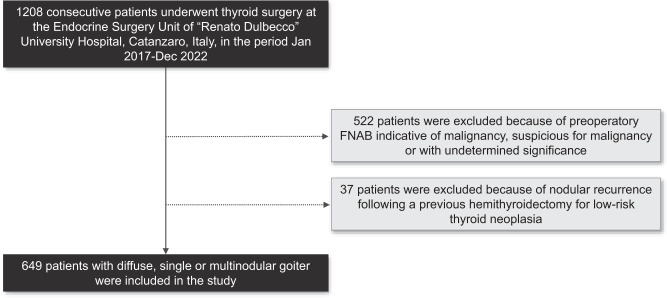
Flow chart indicating the selection of the study population

Between January 2017 and December 2022, a total of 1208 consecutive individuals underwent thyroid surgery at the Endocrine Surgery Unit of the tertiary care “Renato Dulbecco” University Hospital in Catanzaro, Italy. Patients who had received a preoperatory FNAB result indicating malignancy, suspicion for malignancy, or undetermined significance according to the Italian Society for Anatomic Pathology and Cytology (SIAPEC) 2014 criteria [24] were excluded from the study. Additionally, individuals who had experienced nodular recurrence following a previous hemithyroidectomy for low-risk thyroid neoplasia were not included. Ultimately, a retrospective analysis was conducted on 649 patients with diffuse or nodular goiter, regardless of whether it displayed autonomous function (Fig. 1). Patients were offered the option of surgery if they exhibited symptoms related to compression, had a nodule larger than 40 mm, or showed significant growth of a nodule on ultrasound imaging. Prior to surgery, all patients underwent neck ultrasonography, and those with plunging goiter also underwent a chest CT scan. Two or more nodules observed on preoperative ultrasound were defined as MNG, and 557 patients were diagnosed with MNG based on this definition. Additionally, 78 patients were diagnosed with SNG (Fig. 1). Surgery was performed when patients were in a state of pharmacological euthyroidism. Routine measurement of preoperative serum calcitonin levels was not conducted. All patients included in the study were of Caucasian ethnicity and resided in the Calabria region of southern Italy, specifically in the provinces of *Catanzaro*, *Crotone*, *Vibo Valentia* and *Cosenza*. The rural and inland areas of these provinces have been historically characterized by a persistent mild-to-moderate deficiency of iodine and an elevated prevalence of goiter [23]. For the purpose of the study, the following information was collected for each participant: sex, age at thyroid surgery, type of thyroid surgery (total or near-total thyroidectomy vs lobectomy), subtype of ITC based on histopathological features, size of ITC (categorized as <6 mm, 6–10 mm, 11–39 mm, >40 mm in the maximum diameter), presence of extrathyroidal extension (including both minimal and gross extension), presence of multifocality (including both unilateral and bilateral tumor foci), and lymph node metastasis. Despite most of the histological reports being produced prior to the implementation of the 2022 WHO Classification of Thyroid Neoplasms, the results of this study are presented in accordance with this updated classification [25]. As part of this process, the study investigators revisited the diagnosis of ITC by reviewing the original samples. Whenever available, clinical information pertaining to the long-term instrumental and biochemical follow-up monitoring of individuals diagnosed with ITC was also collected, as described elsewhere [3]. The study was carried out in order to ensure the anonymity of the participants and adhere to the guidelines outlined in the Declaration of Helsinki, as well as the principles of Good Clinical Practice. The collection of data was approved by the ethics committee of Regione Calabria Sezione Area Centro (protocol registry no. 343 of 21 November 2019) and was performed by endocrine researchers who had direct access to medical records.

### Statistical analysis

Continuous variables were represented using the mean value along with the standard deviation (SD), while categorical variables were presented as the count and percentage. Percentages were compared using the Fisher’s exact test. Logistic regression analysis, accounting for relevant covariates, was performed to examine the potential relationship between any clinical or instrumental parameter and ITC. The resulting odds ratios (OR) were accompanied by 95% confidence intervals (CI). A significance level of 0.05 was selected for determining statistical significance. Statistical analysis was conducted using the SPSS 22.0 software (SPSS Inc., Chicago, IL, USA).

## Results

### Clinical characteristics of the study population

The mean age ( ± SD) of the study population was 52.9 ± 11.0, as indicated in Table 1. The majority of the participants were female, accounting for 81.8% of total. In 615 patients, thyroid surgery was performed without a preoperative FNAB examination. The reasons for performing thyroid surgery in these cases included the presence of multiple nodular lesions (which were negative for suspicious ultrasound signs), compressive symptoms, aesthetic concerns, or Graves’ disease that did not respond to medical treatment. Prior to surgery, 554 patients were diagnosed with MNG, 81 with SNG, and 14 with Graves’ disease. A preoperative FNAB examination was conducted on 34 patients who had thyroid nodules displaying ultrasound features indicative of potential malignancy, such as irregular or ill-defined margins, hypoechogenicity, presence of microcalcifications, a taller-than-wide shape, solid composition, or a size exceeding 40 mm diameter [24]. The FNAB results showed a benign cytological diagnosis in 32 patients and were inconclusive in 2 patients. Out of the 649 patients included in the study, 436 underwent total or near-total thyroidectomy, while 213 underwent lobectomy. Among those who were incidentally found to have a thyroid carcinoma, the majority initially underwent total thyroidectomy (59 out of 81 cases). Completion thyroidectomy was performed in all other patients who had previously undergone lobectomy and were found to have ITC, including microPTC at risk of local recurrence (i.e., due to multifocality, capsular invasion, and/or extrathyroidal extension). Importantly, no permanent surgical complications were observed in this series of patients.

**Table 1:** Characteristics of study participants and type of thyroid surgery

Characteristics	Mean (SD) or *N* (%)
Age at surgery, yrs	52.9 ± 12.0
Female sex, *N*	531 (81.8%)
Inconclusive preoperative FNAB, *N*	2 (0.3%)
Benign preoperative FNAB, *N*	32 (4.9%)
Missing preoperative FNAB, *N*	615 (94.8%)
Lobectomy^a^, *N*	213 (32.8%)
Total o near-total thyroidectomy, *N*	436 (67.2%)

^a^Includes patients who had received a diagnosis of incidental thyroid carcinoma (ITC) and underwent a second surgical procedure for the completion of thyroidectomy (*n* = 22). Preoperative fine-needle aspiration biopsy (FNAB) results were interpreted according to the Italian SIAPEC 2014 classification

### Histopathological findings and prevalence of incidental thyroid cancer (ITC)

Table 2 presents findings from the histopathological examination. The overall occurrence of ITC was 12.5%. According to the 2022 WHO classification of thyroid neoplasms [25], we observed 2 cases (0.3%) of low-risk follicular cell-derived thyroid neoplasms, including 1 case of non-invasive follicular thyroid neoplasm with papillary-like nuclear features (NIFTP) and 1 case of thyroid tumor of uncertain malignant potential (UMP). Additionally, there were 78 cases (12.0%) of malignant follicular cell-derived neoplasms, consisting of 72 papillary thyroid carcinomas, 5 follicular thyroid carcinomas, and 1 oncocytic carcinoma. We also identified 1 case of medullary thyroid carcinoma (with a maximum diameter of 5 mm). Furthermore, 552 cases (85.8%) of benign follicular cell-derived thyroid tumors were diagnosed, including 478 cases of follicular nodular disease, 67 cases of follicular adenoma, and 6 cases of oncocytic adenoma. Additionally, 16 cases of chronic lymphocytic thyroiditis, typical of Hashimoto’s or Graves’s disease, were observed (Table 2). The prevalence of ITC was similar among patients with SNG (11 out of 78 patients, 14.1%), patients with multinodular MNG (70 out of 557, 12.6%), and patients with Graves’ disease (2 out of 14, 14.3%).

**Table 2:** Histopathological results according to the 2022 WHO Classification of Thyroid Neoplasms

Thyroid histopathology	*N* (%)
Chronic lymphocytic thyroiditis	16 (2.5%)
Benign follicular cell-derived thyroid tumors	72 (85.8%)
• Follicular nodular disease	478
• Follicular adenoma	67
• Oncocytic adenoma	6
Low-risk follicular cell-derived thyroid neoplasms	2 (0.3%)
• NIFTP	1
• UMP	1
Malignant follicular cell-derived thyroid neoplasms	78 (12.0%)
• Papillary thyroid carcinoma^a^	72
• Follicular thyroid carcinoma	5
• Oncocytic carcinoma	1
Medullary thyroid carcinoma	1 (0.2%)

*NIFTP* non-invasive follicular thyroid neoplasm with papillary-like nuclear features, *UMP* thyroid tumor of uncertain malignant potential

^a^Both micro (*n* = 53) and macro (*n* = 19) papillary thyroid carcinomas are included in the final count.

### Incidental papillary thyroid carcinoma (PTC)

Among the 72 patients with incidental PTC, 53 tumors (73.6%) had a maximum diameter of 10 mm or less, thus meeting the criteria for classification as microPTC. The average size of these tumors was found to be 4.5 mm, with 35 of them measuring less than 6 mm. Four of the microPTC cases were found to be multifocal. Of the microPTC cases, 33 (62.3%) exhibited the classic papillary subtype, while the remaining 20 (37.7%) showed the follicular subtype. In most patients with microPTC, the tumor was contained within the nodular capsule, with only 7 cases showing capsular invasion and 3 cases showing extrathyroidal extension. The only significant histopathological difference between microPTC and macroPTC was the absence of lymph node involvement. However, there was a notable trend towards a minor perithyroidal invasion and a higher prevalence of the classic papillary subtype in microPTC, as indicated in Table 3. Interestingly, out of the 14 patients diagnosed with Graves’ disease who underwent thyroidectomy because of ineffective medical intervention, the 2 cases displaying ITC were identified as microPTC.

**Table 3:** Histopathological features of incidental papillary thyroid carcinoma (PTC), stratified into microPTC and macroPTC based on tumor size

	MicroPTC (*n* = 53)	MacroPTC (*n* = 19)	*P* value
Maximum diameter, mm	4.5 ± 2.8	19.3 ± 10.8	–
Maximum diameter <6 mm, *N*	35 (66.0%)	–	–
Maximum diameter 6–10 mm, *N*	18 (34.0%)	–	–
Maximum diameter 11–39 mm, *N*	–	17 (89.5%)	–
Maximum diameter ≥40 mm, *N*	–	2 (10.5%)	–
Multifocality, *N*	4 (7.5%)	4 (21.0%)	0.195
Classic PTC subtype, *N*	33 (62.3%)	7 (36.8%)	0.066
Capsular invasion, *N*	7 (13.2%)	2 (10.5%)	0.999
Extrathyroidal extension, *N*	3 (5.7%)	4 (21.1%)	0.074
Lymph node metastasis, *N*	0.0 (0%)	5 (26.3%)	<0.001

### Predictors of incidental microPTC

Based on the data presented in Table 4, the occurrence of incidental microPTC was found to be higher in women. A preliminary logistic regression analysis conducted on the entire patient population revealed a significant association between being female and having incidental microPTC at a final histopathological examination [OR: 4.083 (95% CI: 1.079–15.448), *p* = 0.038]. This association became stronger when patients with ITC only were included in the analysis [OR: 5.061 (95% CI: 1.238–20.690), *p* = 0.024]. Furthermore, the strength of this association increased further when age was taken into account as a covariate [OR: 5.061 (95% CI: 1.238–20.690), *p* = 0.024]. No significant associations were observed between microPTC and other variables such as the type of nodular disease (MNG vs SNG) or the type of thyroid surgery performed (lobectomy vs total or near-total thyroidectomy), as well as the indication for surgery (presence of compressive symptoms vs toxic or bilateral nodular goiter). The insufficient number of incidental macroPTC cases included in this study resulted in the lack of statistical power for developing a dedicated logistic regression model, thereby preventing the identification of any predictor variables for this specific group of incidental tumors.

**Table 4:** Histopathological features of the incidental thyroid carcinoma (ITC) according to sex

	Female (*n* = 531)	Male (*n* = 118)
MicroPTC, *N*	49 (9.2%)	4 (3.4%)
Malignant follicular cell-derived thyroid neoplasm >10 mm (all subtypes), *N*	20 (3.8%)	5 (4.2%)
Low-risk follicular cell-derived thyroid neoplasm, *N*	1 (0.2%)	1 (0.8%)
Medullary thyroid carcinoma, *N*	0 (0.0%)	1 (0.8%)
ITC (total), *N*	70 (13.2%)	11 (9.3%)

### Postoperative follow-up

The decision regarding postoperative treatment was primarily informed by the histopathological findings. In accordance with current guidelines, patients with T1a (as per the AJCC/TNM stage [3]) follicular epithelial-derived thyroid carcinoma, NIFTP, or UMP were not administered radioiodine metabolic therapy [26]. On the other hand, radioiodine metabolic therapy was recommended for all other patients with T1b-T3a thyroid carcinoma of follicular origin. The mean length of postoperative follow-up was 3.3 ± 1.9 years, with a median duration of 3 years. At present, all patients remain alive and continue to be monitored with neck ultrasonography and biochemical markers of disease (i.e., thyroglobulin). Only one patient with T3a thyroid carcinoma necessitated additional neck surgery due to recurrence of the disease. None of the remaining patients have exhibited any evidence of local recurrence or progression to metastatic systemic disease.

## Discussion

ITC is occasionally observed on histopathological examinations following thyroid surgery for what preoperatively appeared to be a benign disease. The importance of these thyroid tumors, apart from their frequency, lies in their potential aggressive biological behavior. Several studies have investigated the occurrence of ITC in various populations, revealing a wide range of prevalence rates [16, 27]. Our study specifically focused on a genetically homogeneous population from Calabria, southern Italy, with low to moderate iodine deficiency, and found an overall ITC prevalence rate of 12.5%. Additionally, the majority of cases were microcarcinomas, with 53 being microPTC. These findings align with similar studies conducted on different Italian populations [13, 28–30].

MicroPTC has been frequently described as a slow-growing disease, leading to a reconsideration of the traditional practice of immediate thyroid surgery for these types of tumors [8–11]. Current recommendations suggest alternative approaches such as lobectomy or active surveillance, which involves regular imaging studies and thyroglobulin measurements for patients with non-incidental microPTC who do not have known preoperative risk factors [10, 11, 31–33]. However, there is still a limited understanding of preoperative risk factors that can definitively distinguish between low risk and intermediate-high risk microPTC, which would require more aggressive treatment. The advancement in molecular characterization of thyroid cancer is expected to address this uncertainty and provide better guidance for the most appropriate treatment for microPTC [34]. The distinction between incidental and non-incidental microPTC has been a subject of debate in previous studies [35, 36]. However, a recent study found that more than 25% of non-incidental microPTC cases, initially considered to have a low risk of recurrence, actually displayed intermediate-high risk disease after surgery. This resulted in a higher rate of incomplete response during follow-up [37]. Moreover, a recent study has shown that incidental cases of microPTC frequently display characteristics such as multifocality (approximately 15.6%) and bilaterality (approximately 7.2%), as well as hidden lymph node metastasis (up to 57.7%) [38]. Considering these aspects, which are substantially corroborated by our own research findings, we have embraced total thyroidectomy, in specific scenarios as a completion procedure following lobectomy, as the optimal surgical approach for all microPTC patients, even in the absence of apparent lymph node involvement. The administration of radioiodine treatment after surgery has been carried out, as needed, in adherence to the guidelines outlined by the American Thyroid Association (ATA) for all cases of follicular-originated ITC [31]. No instances of local recurrence or systemic disease progression have been observed, except in one patient with macroPTC. Overall, we concur with other authors who argue that total thyroidectomy is the optimal procedure in the presence of MNG [30, 36, 38–40]. Total thyroidectomy is considered a safe procedure when performed by experienced, high-volume, endocrine surgeons and, in certain situations, a less invasive video-assisted approach may be used. This procedure has demonstrated a minimal risk of complications, reduced likelihood of disease recurrence, and enhanced ability to monitor patients through the use of radioactive iodine scans and thyroglobulin measurements [36, 41, 42].

Historically, it was believed that hyperthyroidism caused by Graves’ disease could protect against thyroid carcinoma due to the suppression of thyroid stimulating hormone, which could potentially delay the development of malignancies [28, 43]. However, this historical notion has been challenged by other evidence, which disproves the protective effect of hyperthyroidism against thyroid cancer [44] and instead demonstrates an increased occurrence of thyroid carcinoma in individuals with hyperthyroidism [45]. Nonetheless, a recent meta-analysis focusing on Graves’ disease without thyroid nodules found that the occurrence of thyroid carcinoma was actually a rare event (5%), although this percentage significantly increased when thyroid nodules were also present [46]. In contrast, our surgical series found that 14.3% of patients with Graves’ disease without thyroid nodules had ITC, primarily in the form of microPTC, which is consistent with other reports [28, 29, 36]. However, there is conflicting evidence in the existing literature regarding the occurrence of ITC in Graves’ disease compared to nodular goiter, even if autonomously functioning. Some studies have reported a higher prevalence of ITC in nodular goiter compared to Graves’ disease [47, 48], while others have not observed any difference [36, 49]. Similar to these latter studies, we found a comparable prevalence of ITC in patients with Graves’ disease (14.3%), SNG (14.1%), and MNG (12.6%). The significant presence of ITC in patients with Graves’ disease emphasizes the need for comprehensive diagnostic evaluations and suggests that total thyroidectomy should be considered as a viable treatment option, especially when nodular disease is present. Additionally, there was no discernible difference in the occurrence of ITC between SNG and MNG. Previous research has indicated that MNGs were less likely to be associated with thyroid cancer in comparison to solitary nodules. In line with this, a recent meta-analysis has suggested that solitary thyroid nodules carry a higher risk of ITC compared to MNGs [22]. Nevertheless, this association’s validity and strength are questionable due to the limited quality of the studies available [50].

Many studies have identified patient age, gender, thyroid nodule size, and thyroid gland weight as independent risk factors for the development of thyroid cancer [16, 51, 52]. However, contrasting results have been reported in other reports [28, 48]. Particularly, the association between sex and thyroid cancer has yielded conflicting findings. One recent study found that men had a higher risk of incidental PTC [16], while a more recent study by Bove et al. revealed a higher prevalence of ITC in the female population [53]. Our findings are consistent with the latter study, suggesting that being female was a predictive factor for the presence of incidental microPTC in surgically excised thyroid tissue. Nevertheless, it is important to note that the prevalence of ITC reported by Bove et al. (2.8%) was significantly lower than our observed rate (12.5%). This divergence may be attributed to genetic variations among the populations under investigation [53]. Additionally, it is worth acknowledging that being male has been linked to a worse prognosis in patients with thyroid carcinomas [54–56]. Although the present work does not primarily focus on the prognosis of ITC, previous findings from our own group indicate a higher occurrence of both benign and malignant nodules in Calabria among females compared to males (approximately 3:1 ratio) [24], with males tending to have a higher risk of persistent or recurrent malignant disease in non-incidental tumor cases [3]. No specific sex-related issues have emerged during the postoperative follow-up of ITC in this study, and it remains unclear whether being female could be regarded as a favorable prognostic factor.

A significant limitation of this work is the relatively small sample size of the study population and the fact that it was conducted retrospectively in a single tertiary care surgical center. However, it is important to highlight that the patients included in this study were from a genetically homogeneous population of southern Italy, which helps to minimize potential biases related to genetic factors [3, 24]. Furthermore, the findings regarding the incidence of ITC are in line with those observed in other populations living in different endemic goiter areas. Other drawbacks include the relatively brief period of postoperative follow-up and the limited number of events (with only one case of disease recurrence), both of which hinder the ability to draw definitive conclusions regarding the long-term prognosis of ITC in Calabria.

In conclusion, ITC is a common finding, particularly among women, in patients living in an endemic goiter area who undergo thyroid surgery for apparently benign thyroid diseases. Given that ITC is typically a small cancer, which can be effectively treated with thyroid excision, with or without radioiodine therapy, it is reasonable to recommend close monitoring of patients with MNG to detect thyroid carcinomas at an early stage. In cases where thyroid ablation is deemed necessary, the potential use of total or near-total thyroidectomy as a suitable approach for treating MNG or Graves’ disease should be considered.
